# Activity map of the tammar X chromosome shows that marsupial X inactivation is incomplete and escape is stochastic

**DOI:** 10.1186/gb-2010-11-12-r122

**Published:** 2010-12-23

**Authors:** Shafagh Al Nadaf, Paul D Waters, Edda Koina, Janine E Deakin, Kristen S Jordan, Jennifer AM Graves

**Affiliations:** 1 Research School of Biology, The Australian National University, Biology Place, Canberra, 0200, Australia; 2 ARC Centre of Excellence for Kangaroo Genomics, Research School of Biology, The Australian National University, Biology Place, Canberra, 0200, Australia; 3 Current address: Cytogenetics Department, ACT Pathology, The Canberra Hospital, Yamba Drive, Canberra, 2605, Australia

## Abstract

**Background:**

X chromosome inactivation is a spectacular example of epigenetic silencing. In order to deduce how this complex system evolved, we examined X inactivation in a model marsupial, the tammar wallaby (*Macropus eugenii*). In marsupials, X inactivation is known to be paternal, incomplete and tissue-specific, and occurs in the absence of an XIST orthologue.

**Results:**

We examined expression of X-borne genes using quantitative PCR, revealing a range of dosage compensation for different loci. To assess the frequency of 1X- or 2X-active fibroblasts, we investigated expression of 32 X-borne genes at the cellular level using RNA-FISH. In female fibroblasts, two-color RNA-FISH showed that genes were coordinately expressed from the same X (active X) in nuclei in which both loci were inactivated. However, loci on the other X escape inactivation independently, with each locus showing a characteristic frequency of 1X-active and 2X-active nuclei, equivalent to stochastic escape. We constructed an activity map of the tammar wallaby inactive X chromosome, which identified no relationship between gene location and extent of inactivation, nor any correlation with the presence or absence of a Y-borne paralog.

**Conclusions:**

In the tammar wallaby, one X (presumed to be maternal) is expressed in all cells, but genes on the other (paternal) X escape inactivation independently and at characteristic frequencies. The paternal and incomplete X chromosome inactivation in marsupials, with stochastic escape, appears to be quite distinct from the X chromosome inactivation process in eutherians. We find no evidence for a polar spread of inactivation from an X inactivation center.

## Background

In therian mammals (eutherians and marsupials), the sex of an embryo is determined by the presence or absence of a Y chromosome, whereby males have a Y and a single X, and females have two X chromosomes. The eutherian X and Y chromosomes show homology within a pseudoautosomal region that pairs at meiosis, and most Y genes have a homologue on the X chromosome, from which they clearly evolved. This supports the hypothesis that the X and Y evolved from an ordinary autosome pair via degradation of the Y, after it acquired a testis-determining factor, *SRY *(reviewed in [1]).

The sex chromosomes of eutherian and marsupial mammals share extensive homology, although the marsupial sex chromosomes lack the autosomal added region that was added to the eutherian X and Y [1], so are smaller than those of eutherian mammals. The marsupial X and Y are completely differentiated; there is no pseudoautosomal region, and the marsupial X and Y show no homologous pairing at male meiosis [2]. However, all but one gene on the marsupial Y have diverged partners on the X (Murtagh VJ, Sankovic N, Delbridge ML, Kuroki Y, Boore JL, Toyoda A, Jordan KS, Pask AJ, Renfree MB, Fujiyama A, Graves JAM & Waters PD, submitted).

Since most X genes were originally present on the proto-Y chromosome, the progressive loss of Y gene function resulted in a dosage imbalance of X-borne genes between XX and XY individuals. This disparity of X gene expression between the sexes is thought to have resulted in the evolution of a dosage compensation mechanism.

An effective way to understand the evolution of dosage compensation mechanisms is to study dosage compensation in distantly related groups of mammals and non-mammal vertebrates. Mechanisms that are shared by different species are likely to have been present in a common ancestor, whereas features that are lineage-specific were probably acquired after the species diverged.

X chromosome inactivation (XCI) appears to be a mammal-specific dosage compensation mechanism, since the bird Z chromosome does not undergo a whole-chromosome inactivation [3], and Z-borne genes display incomplete and locus-specific dosage compensation [4] and biallelic expression [5,6]. Surprisingly, this partial and variable dosage compensation seems to be shared by monotremes, the most basal mammal group [7]. The egg-laying monotremes have a complex of serially translocated sex chromosomes [8,9] that share no homology to the sex chromosome of other (therian) mammals, but instead have homology to the ZW sex chromosomes of birds [10]. In monotremes, genes are transcribed from both X chromosomes in the cell population. Dosage compensation for each gene is achieved by transcription from only one of the two alleles in a characteristic proportion of cells [7].

Marsupial mammals, however, do appear to share XCI with eutherians, as shown by early isozyme studies (reviewed in [11]). Since X chromosomes of eutherians and marsupials are largely homologous, it is expected that the XCI mechanisms of the two groups also share a common evolutionary history.

In eutherians, XCI occurs early in female embryonic development. It is controlled *in cis *by a master regulatory locus, *XIST *(X inactive specific transcript), within an X inactivation center, which transcribes a non-coding RNA [12]. The choice of which parentally derived X chromosome becomes inactive is random in the embryo proper, but paternally imprinted in extraembryonic membranes in at least rodent and cow [13-17]. Several epigenetic modifications maintain the heterochromatic and transcriptionally silenced state of the eutherian inactive X chromosome (Xi) throughout the cell cycle (reviewed in [18]).

In contrast to the stable and complete XCI system of eutherians, marsupial XCI appears to be incomplete, locus- and tissue-specific (reviewed in [19]). Decades-old studies of three X-borne genes in two kangaroo species, using isozymes, revealed that in marsupials the allele on the maternally derived X is always active, and the paternally derived allele chromosome is inactivated. Nonetheless, some loci on the paternal X escape inactivation to various extents in many tissues, including cultured fibroblasts, and the suggestion was made that escape is controlled in a polar fashion from an inactivation center [20]. However, the diverse methodologies and different species used, and the limited number of polymorphic genes available, made it difficult to decipher the mechanism of marsupial XCI (reviewed in [19]).

The molecular mechanism of XCI in marsupials shares some features with that of eutherian XCI, including late DNA replication and loss of histone marks associated with transcriptional activity [21,22]. Yet there are major differences in the molecular mechanism of XCI in eutherians and marsupials. Perhaps the most significant is the absence of the *XIST *gene in marsupials, implying that the regulation of imprinted XCI in marsupials is achieved by an *XIST*-independent method [23,24]. The apparent absence of differential DNA methylation at CpG islands [25-27] suggests that maintenance of inactivation is achieved differently in marsupials and eutherians.

Significantly, paternal XCI was discovered later to occur also in rodent extraembryonic tissues, leading to the suggestion that marsupials represent an ancestral and simpler XCI regulation system, to which layers of molecular complexity were added during eutherian evolution [28]. This idea is supported by the observations that, like marsupial XCI, paternal XCI in mouse extraembryonic tissues is less stable, incomplete and does not involve DNA methylation [29]. Furthermore, features that were once thought to be specific to marsupial XCI, such as the incomplete inactivation of the X, have parallels in the discovery of many genes on the human X that escape XCI [30].

It therefore becomes essential to answer fundamental questions about marsupial XCI, including the extent to which different genes are inactivated, whether control of inactivation is locus-specific, regional or chromosome wide, and whether marsupial XCI initiates from a yet undiscovered inactivation center. Moreover, it is important to know whether the incomplete inactivation observed for some genes in fibroblasts is the result of all cells in a fibroblast population expressing maternal and paternal alleles differently, or of different ratios of cells in the population expressing from either one or both X chromosomes.

To answer these questions it was necessary to investigate XCI at the cellular level, rather than observing the population average by biochemical approaches used previously with whole cell lysates. We therefore examined the expression status of 32 X-borne loci using RNA-fluorescence *in situ *hybridization (FISH). Surprisingly, RNA-FISH of each locus produced a reproducible (between experimental and biological replicates) frequency of 1X-active and 2X-active nuclei. Loci on one X (the active X, Xa) were coordinately expressed in every cell, but loci on the other X (the inactive X, Xi) were independently expressed at locus-specific frequencies, suggesting that escape from inactivation is controlled at the level of the probability, rather than the amount, of transcription from the inactive X. The activity profile of the marsupial X revealed no correlation between gene location and XCI status, implying that there is no regional control of XCI and, therefore, no XCI center, and was unrelated to the presence of a Y-borne allele.

## Results

We chose to examine XCI in the tammar wallaby, *Macropus eugenii*, the Australian model kangaroo, whose genome has recently been sequenced and a detailed physical map constructed [31]. We first gained an overall assessment of the level of XCI by comparing the expression of 13 X-borne genes in male- and female-derived fibroblasts using quantitative PCR (qPCR). We then determined the frequency of escape from XCI in individual nuclei using RNA-FISH, which allowed us to construct an activity map of the tammar wallaby X.

### Determination of female:male expression ratios by qRT-PCR

**Figure 1:**
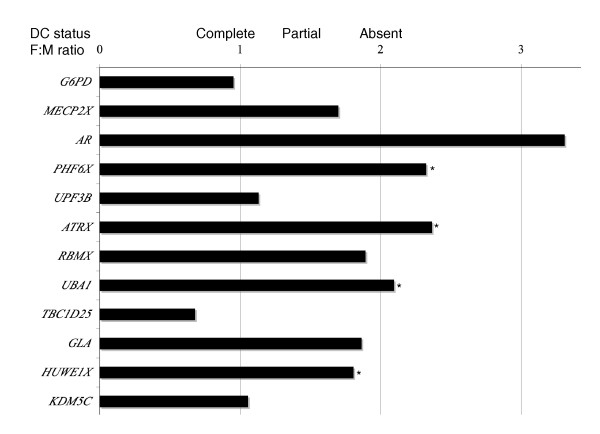
**Female:male ratio for average expression of tammar X-borne genes in fibroblast cells (five males, six females) normalized to the autosomal *GAPDH *housekeeping gene**. Genes are presented in the order in which they are located on the X, from the centromere down. Ratios varied between complete compensation (ratio 1.0) and no compensation (ratio 2.0). *, statistically significant association (*P *< 0.05).

Since there is no quantitative data on the extent of dosage compensation for any X-borne gene in the tammar wallaby, we first used qPCR to examine the expression of 13 genes in 5 male- and 6 female-derived fibroblast cell lines (Figure 1; Additional file 1). For genes with Y-borne homologues, we used primers that specifically amplified the X-borne locus. Although the considerable variability between individuals made quantitative analysis difficult, the female to male ratios for different genes ranged from 1 to 3, suggesting that X-borne genes are incompletely compensated to different extents. The ratios were unrelated to the presence or absence of a Y-borne paralogue. This suggests remarkable heterogeneity in transcriptional inactivation of X-borne genes in female marsupial cells.

### RNA-FISH detection of primary transcript

The XCI status of X-borne genes was examined using RNA-FISH, which permits detection of primary transcripts in interphase nuclei by hybridization with large probes (BACs or fosmid clones in this study) containing introns that are spliced out from cytoplasmic mRNA.

**Figure 2:**
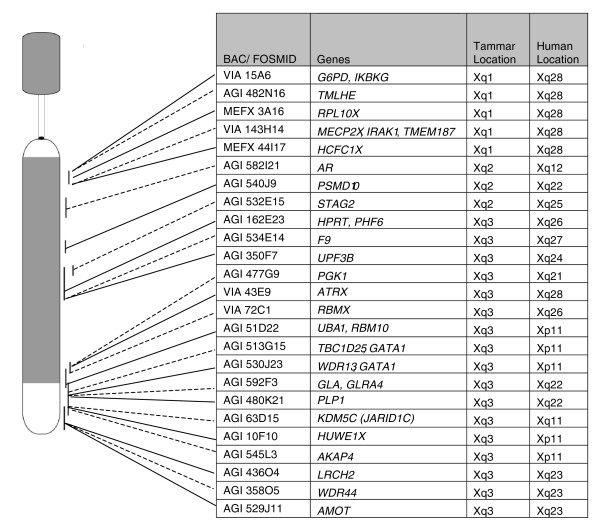
**Physical map of the tammar wallaby X chromosome showing location of analyzed genes**. Locations of BACs and fosmids used for RNA-FISH on the tammar X chromosome. The DAPI dense regions are indicated in grey. BAC and fosmid clones used in this study and the genes they bear, genome coordinates and the band location of human orthologues are shown.

We selected 25 X-borne probes, cloned from the tammar wallaby X chromosome, 18 of which contained a single gene, and 7 of which contained 2 or more genes. These probes represented 32 genes distributed along the length of the wallaby X chromosome (Figure 2). For the BACs containing more than one gene, hybridization to transcript from any constituent gene within the locus assayed will be observed as a single signal. Chosen genes all have orthologues on the human X chromosome that are distributed over every chromosome band in the X conserved region (Figure 2).

In interphase female-derived cells, nuclei expressing a gene (or at least one gene in a multigene BAC) from only one of the two X chromosomes (1X-active) were observed as a single signal, whereas cells expressing a gene from both X chromosomes (2X-active) were observed as two signals within a nucleus.

### Efficiency and specificity of RNA-FISH in fibroblast cells

**Figure 3:**
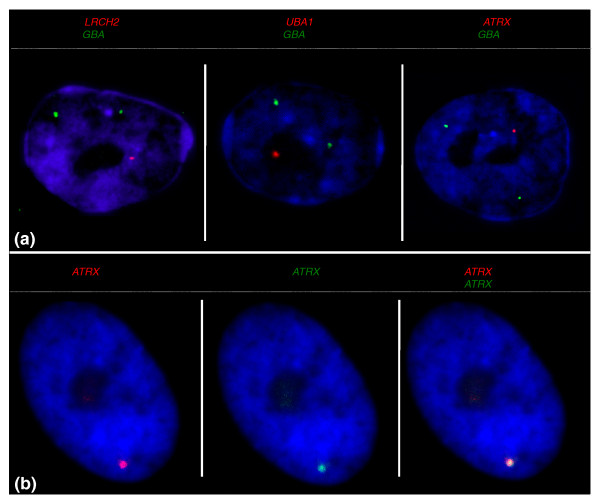
**Transcriptional activity of an X-borne gene and autosomal control in male fibroblasts**. Loci are color coded above panels. **(a) **Male fibroblast nuclei with transcription from two autosomal *GBA *alleles (green) and the single X-borne locus (red). **(b) **Analysis of *ATRX *by sequential RNA-DNA FISH. Merged panel reveals that the RNA (red) and DNA (green) FISH signals co-localize with no cross-hybridization to the Y paralogue. Nuclei are counterstained with DAPI (blue).

We first assessed efficiency and specificity of hybridization for each probe using male-derived fibroblasts. In male nuclei (XY), a single signal is expected for an X-borne gene probe. To control for polyploidy and the accessibility of cells to probe hybridization, we designed two-color RNA-FISH experiments with a probe containing X-borne gene(s), and a second probe (Me_KBa 206L23) containing an autosomal control gene (*GBA *located on tammar chromosome 2). The two probes were labeled with different fluorochromes and co-hybridization was carried out for each locus in male interphase nuclei. At least 100 nuclei having two *GBA *signals were scored for each X gene (Figure 3a, Table 1).

**Table 1:** Quantitative analysis of male fibroblast RNA-FISH data

Genes on BACs or fosmids	Percent male nuclei with one signal
***G6PD*, *IKBKG***	95%
*TMLHE*	96%
*RPL10X*	98%
***MECP2X*, *IRAK1*, *TMEM187***	99%
*HCFC1X*	99%
*AR*	94%
*PSMD10*	98%
*STAG2*	95%
*HPRT*, *PHF6X*	95%
*F9*	0%
*UPF3B*	99%
*PGK1*	98%
***ATRX***	98%
*RBMX*	95%
*UBA1*, *RBM10*	98%
*TBC1D25*, *GATA1*	98%
*GATA1*, *WDR13*	94%
*GLA*, *GLRA4*	98%
*PLP1*	0%
*KDM5C*	96%
***HUWE1X***	97%
*AKAP4*	99%
*LRCH2*	96%
*WDR44*	94%
*AMOT*	95%

Frequency of nuclei with single signal for X-borne loci investigated in this study. The efficiency of RNA-FISH hybridization for each locus is based on at least 100 male nuclei with two signals for the control autosomal gene, and therefore diploid. Genes highlighted in bold were used in sequential RNA-DNA FISH experiments.

We calculated the efficiency of hybridization from the frequency of diploid nuclei showing a single signal for the test gene. This frequency was between 95% and 98% for all loci except *F9 *and *PLP1*, which were evidently not expressed in male and female marsupial fibroblasts, and were eliminated from the analysis (Table 1). No diploid cells had more than a single signal for the test gene. For each experiment only a few nuclei (fewer than 6%) showed an absence of both test and control signals, which we attributed to shielding of target sequences in some cells.

Some of our X-borne genes have Y-borne paralogues, shown by DNA-FISH using both X-derived and Y-derived BACs to have diverged beyond recognition (Murtagh VJ, Sankovic N, Delbridge ML, Kuroki Y, Boore JL, Toyoda A, Jordan KS, Pask AJ, Renfree MB, Fujiyama A, Graves JAM & Waters PD, submitted) [31]. These genes, too, showed only a single site of transcription for the test gene. In order to be quite certain that the probes detected only the X-borne gene, we also conducted sequential RNA-DNA FISH for four X-borne probes with Y paralogues in male fibroblasts. A single DNA-FISH signal was observed in every male nucleus. The RNA-FISH analysis of all four genes detected a single signal, which co-located to the site of the DNA-FISH signal (Figure 3b). This lack of cross-hybridization between X and Y paralogues meant that we could be confident that the X-probe detected only the X-borne locus.

### One X chromosome is maintained active in all female cells

**Figure 4:**
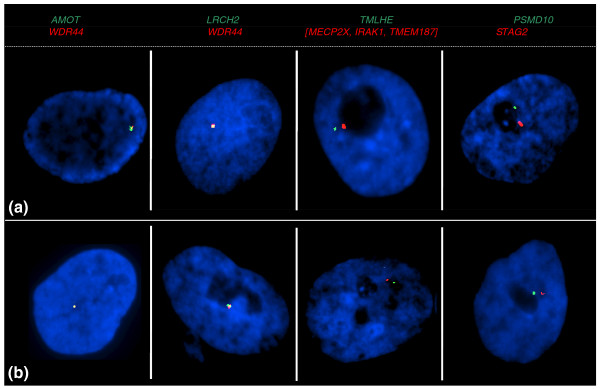
**Coordinate transcriptional activity of neighboring X-borne loci assayed by two-color RNA-FISH in male and female fibroblasts**. Loci are color coded above panels. **(a) **Male nuclei with transcription from two X-borne loci on the single X chromosome. **(b) **Female nuclei with transcription from two X-borne loci on the active, but not the inactive, X chromosome. Nuclei are counterstained with DAPI (blue).

In order to determine whether transcription from one of the two X chromosomes of females is coordinately regulated, we performed RNA-FISH using probes for two neighboring X-borne loci labeled with different colored fluorochromes. As a control, co-hybridization was carried out in male interphase nuclei (Figure 4a).

In male cells, RNA-FISH signals from neighboring loci were expected to co-locate within the nucleus, and their distances apart could be observed. In female cells, the two signals were expected to co-locate at this same distance when transcribed from the same X chromosome, but would be further apart if transcribed from different X chromosomes. For loci lying far apart on the X the arrangement of signals was difficult to interpret. We therefore tested simultaneous expression of four pairs of X-borne probes that were located sufficiently close together on the tammar X chromosome to give unambiguous results (Figure 4).

Female fibroblasts were tested, and 100 cells analyzed that showed a single signal for each locus scored. For each of the four gene pairs, the distance between signals observed in female nuclei was equivalent to the distance in all male cells. This result demonstrated that loci on a single X chromosome are coordinately active, rather than active on different X chromosomes (Figure 4b). This suggests a whole X mechanism that ensures expression of genes from the same active X chromosome (Xa).

#### Escape of loci on the tammar Xi

Our demonstration that the Xa is coordinately controlled used nuclei in which two loci were both expressed from only one X chromosome. However, we observed many diploid nuclei in which loci were expressed from both X chromosomes, suggesting that some or all marsupial genes may escape inactivation on the Xi to some extent, as suggested by our qPCR results.

**Figure 5:**
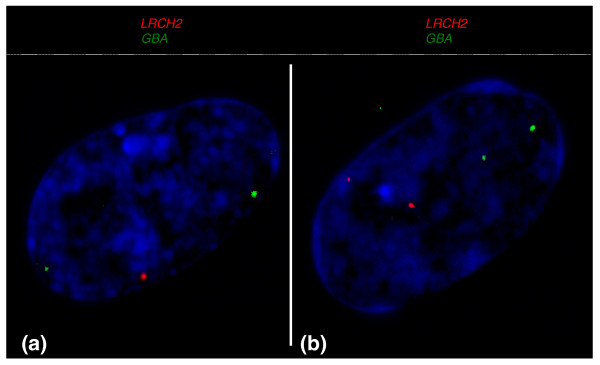
**Transcriptional activity of an X-borne gene and autosomal control in female fibroblasts**. *LRCH2 *(red signal) is on the X and *GBA *(green signal) is on chromosome 2. **(a,b) **Female fibroblast nucleus shows transcription from both autosomal *GBA *alleles (green), and either one (a) or two (b) X-borne *LRCH2 *alleles (red). Nuclei are counterstained with DAPI (blue).

To test for this possibility, we established the frequency of escape from inactivation (expression from both X chromosomes) by performing two-color RNA-FISH experiments with a probe for the test X-borne loci and the autosomal control *GBA *(Figure 5). For a total of 23 loci, we scored the frequency of 1X-active and 2X-active nuclei in at least 100 diploid nuclei (Table 2).

**Table 2:** Quantitative analysis of female fibroblast RNA-FISH data

	Percent female nuclei with		
	
Genes on BACs or fosmids	2 signals	1 signal	0 signals
***G6PD*, *IKBKG***	17.0	82.0	1.0
*TMLHE*	29.0	68.0	3.0
*RPL10X*	27.0	71.0	2.0
***MECP2X*, *IRAK1*, *TMEM187***	41.0	54.0	5.0
*HCFC1X*	7.0	91.0	2.0
*AR*	9.0	87.0	4.0
*PSMD10*	6.7	93.3	0.0
*STAG2*	6.7	92.0	1.3
*HPRT*, *PHF6X*	23.5	73.5	3.1
*UPF3B*	11.7	86.7	1.7
*PGK1*	18.5	80.4	1.1
***ATRX***	59.8	39.2	1.0
*RBMX*	11.0	87.0	2.0
*UBA1*, *RBM10*	67.8	32.2	0.0
*TBC1D25*, *GATA1*	14.3	84.7	1.0
*GATA1*, *WDR13*	14.9	81.3	3.7
*GLA*, *GLRA4*	7.5	89.2	3.2
*KDM5C*	35.0	65.0	0.0
***HUWE1X***	15.0	85.0	0.0
*AKAP4*	44.1	51.0	4.9
*AMOT*	23.0	71.7	5.3
*LRCH2*	5.2	92.8	2.1
*WDR44*	14.3	81.6	4.1

Frequency of nuclei with 2, 1 or 0 signals for each X-borne locus. At least 100 nuclei were scored. Only nuclei with two signals for the autosomal control gene, and therefore diploid, were scored. Male efficiencies were used to calculate the expected frequency of nuclei with two signals, one signal and no signal for each test gene. Expected frequencies of cells with two signals were in excess of 90% for all loci, and observed frequencies were significantly different from these expected frequencies in every case (Chi-square test with 2 degrees of freedom). Genes highlighted in bold were used in sequential RNA-DNA FISH experiments.

All loci tested appeared to escape XCI to some extent, since they were expressed from both X chromosomes in many female nuclei. However, escape was not complete; for all loci, the frequencies of nuclei with a single signal were far greater than would be expected (between 2 and 9%) merely from inefficiency of hybridization, which was measured on male fibroblasts for each experiment (Table 2).

There were no loci that were 1X-active in every cell, and no loci that escaped inactivation in every cell. Rather, within a population of cells each locus had a characteristic frequency in which one or both alleles were expressed. The frequency of 2X-active nuclei ranged from 5% of nuclei for *LRCH2*, representing a locus almost completely subject to inactivation, to 68% for a BAC containing *UBA1 *and *RBM10*, representing a locus largely escaping inactivation (Table 2).

For the loci we tested, six were 2X-active in ≤9% of nuclei (representing almost complete inactivation). Another 11 loci were expressed from both Xs in 11 to 35% of nuclei. In addition, two BACs (containing *AKAP4 *and [*MECP2X*, *IRAK1*, *TMEM187*]) were expressed from both Xs at frequencies of 44% and 41%, respectively. These loci appear to be escaping inactivation in a significant fraction of cells, so are only partially inactivated.

Almost complete escape from inactivation was observed for two of the X-borne BACs, one containing *ATRX *and the other containing *UBA1 *and *RBM10*. These BACs exhibited the highest frequency of 2X-active expression (60% and 68% of nuclei, respectively; Table 2).

Thus, for different loci, different proportions of nuclei are expressed from one or both X chromosomes, suggesting that partial dosage compensation in marsupials is the result of the frequency of 1X-active and 2X-active nuclei in a population of cells, rather than a uniformly lower level of transcription from the Xi over the population of cells. The different XCI patterns observed for different genes suggest that each locus has a characteristic probability of 1X-active or 2X-active expression.

**Figure 6:**
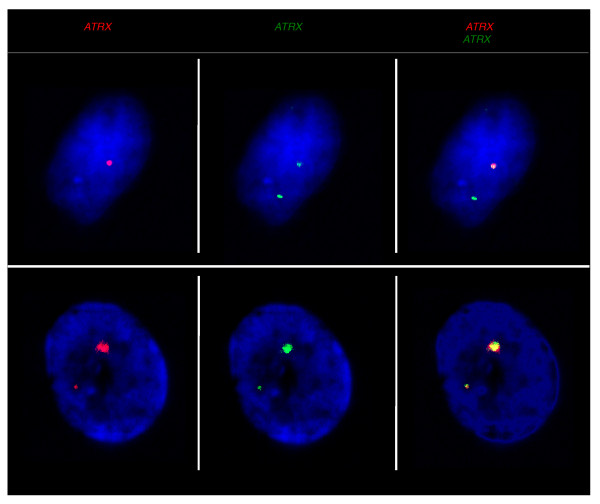
**Expression and localization of *ATRX *by RNA-DNA FISH in female fibroblast nuclei**. **(a,b) **Sequential *ATRX *RNA (red) and DNA (green) FISH reveals that either one (a) or two (b) RNA-FISH signals co-localize with the DNA signals. Nuclei are counterstained with DAPI (blue).

To confirm our observation that the population of female cells included both 1X-active and 2X-active nuclei, we conducted sequential RNA-DNA FISH for four X-borne BACs to control for both the probe accessibility and check that the locus was the site of transcription (Figure 6). The RNA-FISH analysis of all four genes detected nuclei with both 1X-active and 2X-active gene expression in female fibroblast cells from the same individual (Figure 6). Since the DNA-FISH step diminished the RNA signal, the efficiencies of RNA signal hybridization were too low to score the frequency of 1X-active and 2X-active nuclei.

RNA-FISH results were validated for a subset of genes (Additional file 2) on four independently derived primary fibroblast cell lines from different individuals (two male and two female). For each probe, there was little variation between individuals in the frequency of 1X-active and 2X-active nuclei. Thus, each probe produced a characteristic frequency of 1X-active and 2X-active expression, which was reproducible between experimental and biological replicates. We used these frequencies to make an activity map of the Xi.

#### Activity map of the tammar inactive X chromosome reveals no X inactivation center

**Figure 7:**
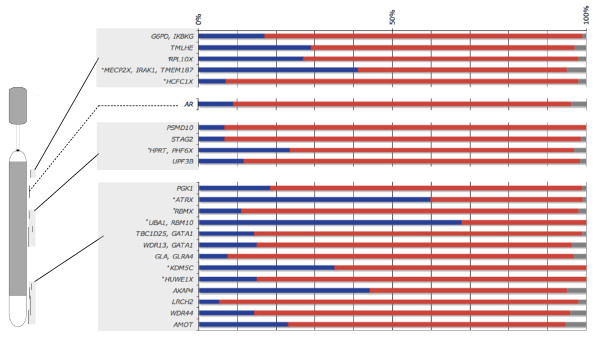
**X chromosome activity map in tammar wallaby female fibroblasts**. RNA-FISH activity map of the tammar wallaby X chromosome. Bars represent percentage of nuclei transcribing from 2 (blue), 1 (red) or 0 (grey) loci. The absence of polarity suggests that no inactivation center co-ordinates inactivation. *X genes with known Y paralogues.

We created an activity map of genes on the tammar X (Figure 7) to determine if there was local, regional or chromosome-wide control of XCI in marsupials that, as for eutherians, spreads from an inactivation center. The 23 loci in this study have been physically mapped and ordered on the tammar X [31].

The map revealed no clustering of loci with either a particularly high or a particularly low frequency of inactivation. For instance, loci that are 2X-active in more than 50% of nuclei ([*UBA1*, *RBM10*] and *ATRX) *are separated by loci with low frequencies of escape from inactivation. These results are inconsistent with the predictions of co-ordinate down-regulation of the whole inactive X chromosome, or of any large X region, and identify no region that might serve as an XCI control center.

#### Escape from inactivation is independent of the presence of a Y paralogue

**Figure 8:**
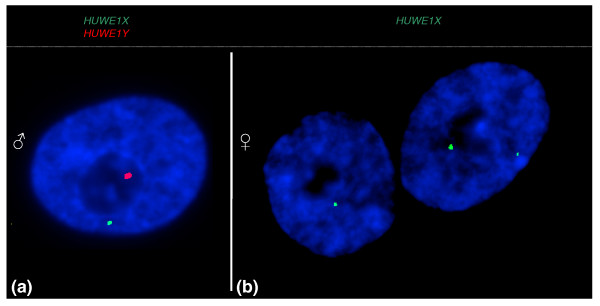
**Transcriptional activity of an X-borne gene and its Y paralogue in male and female fibroblasts**. The *HUWE1Y *probe (red signal) detects the paralogue located on the Y, and the *HUWE1X *probe (green signal) detects the paralogue on the X chromosome. **(a) **Male nucleus with transcription from the single X locus (*HUWE1X*, green) and the single Y locus (*HUWE1Y*, red). Different signal intensities from different probes does not correlate to transcription level. **(b) **Female fibroblast nuclei with transcription from one (left) and two (right) X-borne loci (*HUWE1X*, green), and no expression detected with the Y-specific probe (*HUWE1XY*, red). Nuclei are counterstained with DAPI (blue).

Human X-borne genes that have paralogues on the Y are largely exempt from inactivation, suggesting that the Y copy complements the X, now or in the recent evolutionary past. To investigate a possible relationship between dosage compensation and Y paralogue activity in marsupials, we therefore tested expression from the X- and Y-borne paralogues by two-color RNA-FISH, using differentially labeled probes to the X and Y paralogues. These experiments were carried out for five X-borne genes and their Y paralogues using female and male interphase nuclei (Figure 8, Table 3).

**Table 3:** Y paralogue expression contrasted with X-copy dosage compensation status

Gene name	Y copy expression	Escape from Xi
*ATRX/ATRY*	Not expressed	>50%
*RMBX/RBMY*	Not expressed	0-25%
*PHF6X/PHF6Y*	Not expressed	0-25%
*HUWE1X/HUWE1Y*	Expressed	0-25%
*RPL10X/RPL10Y*	Expressed	25-50%
*HCFC1X/HCFC1Y*	Expressed	0-25%
*MECP2X/MECP2Y*	Expressed	25-50%

As expected, female nuclei showed either one or two signals from the X probe and no signal from the Y probe (Figure 8). In male cells, a single signal was observed from the X and a different colored signal from the Y paralogue, consistent with previous demonstrations of the poor homology between X and Y paralogues (Figure 8). BACs containing *ATRY *and *RBMY*-*PHF6Y *showed signal in <5% of male nuclei tested (Table 3), implying that these genes are not expressed in male fibroblasts. All other Y-borne genes tested were expressed in male fibroblasts (Table 3). No correlation was observed between the presence of a Y paralogue and dosage compensation status of the X-copy. We therefore concluded that the presence of a Y paralogue was neither necessary nor sufficient for escape from inactivation.

#### Escape from inactivation is not coordinated

Our finding that different genes have different frequencies of escape, and that there is no polarity in frequency of expression over the X, still leaves open the possibility that coordinate control operates to regulate expression of genes in smaller domains on the Xi. To test for this possibility, we examined escape from inactivation simultaneously for two X-borne genes that are located close together on the tammar X chromosome and have similar escape frequencies.

**Figure 9:**
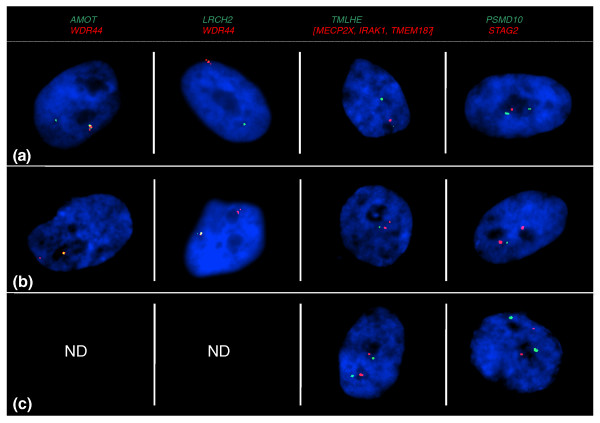
**Two-color RNA-FISH in female fibroblasts reveals independent escape from inactivity of two neighboring X-borne loci**. Loci are color coded above panels. (a) Nuclei in which one gene (green) is expressed from both alleles and the second gene (red) is expressed from only one allele. **(b) **Nuclei in which one gene (green) is expressed from one allele and the second gene (red) is expressed from both alleles. **(c) **Nuclei in which both genes are expressed from both alleles. ND, no nuclei were observed in this category. Nuclei are counterstained with DAPI (blue).

We performed RNA-FISH using two BACs that were labeled with different fluorochromes (Figure 9). These were co-hybridized to male and female fibroblasts. For each comparison, we scored 100 female nuclei in which at least one of the two test loci was expressing from both X chromosomes (Table 4). The hypothesis that genes coordinately escape on the Xi predicts that red and green signals would be present or absent together on the second X chromosome in most nuclei (that is, concordant). However, if silencing of the two genes on the Xi were independent, we would expect to find most nuclei with either one green signal, or one red signal, on the Xi (that is, discordant). For instance, for the gene pair *PSMD10*/*STAG2*, where the frequency of escape is 6.7% for each gene, the hypothesis of independent escape predicts only one nucleus (of the 100 sampled with at least one escaper) escaping at both loci, and 99% of nuclei escaping at one or the other locus. In contrast, the hypothesis of co-ordinate control would predict that nearly all the 100 nuclei sampled should show escape at both loci, and none would be discordant. Similar predictions can be made for each gene pair, although the expected frequencies differ for different pairs of loci, since they have different frequencies of escape.

**Table 4:** Frequency of nuclei expressing one or both of two neighboring X-borne loci (A and B) from the inactive X assayed by two-color RNA-FISH in female fibroblasts

	Percentage nuclei with signal on Xi (*n *= 100)			
	
Gene *A*/gene *B*	*AMOT*, *WDR44*	*LRCH2*, *WDR44*	*TMHLE*, [*MECP2X*, *IRAK1*, *TMEM187*]	*PSMD10*, *STAG2*
-/+	60	27	39	57
+/-	40	74	36	42
+/+	0	0	24	1

Plus signs indicate expressed, dashes not expressed.

For each gene pair, we found that most or all nuclei expressed the two markers discordantly (Figure 9, Table 4). For example, *PSMD10 *and *STAG2 *were expressed discordantly in 99 cells, and coordinately in only one cell (Figure 9c). This suggests that the two genes on the Xi escaped inactivation independently.

Only one pair of loci (*TMLHE*, [*MECP2X*, *IRAK1*, *TMEM187*]) showed a relatively large number of nuclei (24 out of 100) with escape of both loci. Although the observed frequency of concordant escape is greater than the 12% predicted by the hypothesis of independent escape, it is still much lower than the 35% expected of concordance escape.

These results suggest that most pairs of genes, even those located close together, escape inactivation at a different frequency and independently of its neighbor. However, it remains possible that for some gene pairs, escape may be a property of the chromatin domain in which they lie.

## Discussion

Data from venerable isozyme studies show that dosage compensation in XX females is achieved through inactivation of one X chromosome in marsupial, as well as eutherian, mammals. However, unlike the random X inactivation in humans and mice, XCI was found to be paternal in all marsupial species, and at all loci tested. Observation that some genes on the paternal X are fully or partially expressed at the protein level in some kangaroo tissues led to the conclusion that marsupial XCI is incomplete and tissue specific (reviewed in [19]). It is difficult to generalize these findings to the whole X chromosome, or other marsupials, because the results are based on only three genes that were polymorphic in just one or a few marsupial species (not including our model kangaroo, the tammar wallaby).

The availability of a robust physical map of the tammar X chromosome [31], and of the tammar DNA sequence (tammar genome project, in preparation), allowed us to construct an activity map of the whole X chromosome in fibroblasts of the tammar wallaby to test the generality of the old data, and to explore outstanding questions of control of marsupial XCI at the molecular level. We used qPCR to compare the level of expression of several X-borne loci in male- and female-derived fibroblasts, finding that the female:male ratio was different for different genes, but that most genes were more highly expressed in females than in males.

Our most surprising findings were made using RNA-FISH to quantify inactivation on an individual cell basis. This method gave unique information in a species in which few polymorphisms in X-borne genes have been identified. The RNA-FISH was extremely efficient at all loci, detecting expression of 94 to 99% of loci in male cells.

### Marsupial XCI is regulated at the transcriptional level

Investigations of inactivation at the protein level left open the question of whether XCI in marsupials was at the transcriptional level, as it is in eutherians [32]. The present study shows that XCI control is exerted at the transcriptional level also in marsupials, for RNA-FISH revealed that most female nuclei showed only a single signal typical of 1X-active cells. This result is confirmed by the absence of RNA polymerase from the inactive X chromosome (Chaumeil J, Waters PD, Koina E, Gilbert C, Robinson TJ & Graves JAM, submitted).

### Expression from one X chromosome is coordinately controlled

Co-location of signals from neighboring genes in female fibroblast RNA-FISH experiments led us to conclude that genes are coordinately transcribed from the same active X chromosome. For instance, we found that *STAG2 *and *PSMD10 *were co-expressed in all nuclei that showed single-active expression for each locus, demonstrating that genes located close together on the same X are coordinately expressed. Pairwise comparisons using different combinations of other genes showed that all genes tested were active on the same active X chromosome, Xa. We have no way of determining the parental origin of this active chromosome, but all previous investigations on populations of cells have shown that the maternal allele is always expressed, and the inactive allele always comes from the paternal X. We therefore conclude that all alleles on the maternal X are expressed in all cells.

### Expression from Xi is incomplete, and locus specific

We used RNA-FISH to examine expression of loci distributed along the tammar wallaby X chromosome. We found that all genes escaped inactivation to some extent; the percent of escape from inactivation (that is, percent of 2X-active cells) for different genes varied between 5 and 68%. Each locus displays a different frequency of escape, consistent between animals, which implies that escape is locus specific. This partial, locus-specific escape confirmed the preliminary indication from qPCR data that the female:male ratio of the X gene transcript varied from complete dosage compensation to complete escape. This greatly extends the findings from isozyme studies that paternal *PGK1 *and *G6PD *are partly expressed in kangaroo fibroblasts [28,33].

### Escape from marsupial XCI is stochastic

Early studies of partial inactivation at the protein level [34] included the demonstration that single cell clones maintained the same level of paternal expression as the entire population. This was interpreted to mean that partial expression amounted to uniform down-regulation of expression of the paternal allele in all cells. Our qRT-PCR of female:male expression ratios also indicated variable degrees of transcriptional silencing in female cells. However, neither technique applied to populations of cells can distinguish between partial expression due to down-regulation of transcription from the Xi in every cell, or from different frequencies of cells with 1X-active and 2X-active expression.

Our ability to detect transcription at the level of a single nucleus using RNA-FISH therefore allowed us to discover that control is not exerted by down-regulation of the paternal allele in all cells, as had been expected. Rather, the overall level of transcription is regulated by the frequency of nuclei in which the allele on the inactive X is expressed. Regulation appears to be a stochastic (probabilistic) process since different genes show a characteristic frequency of 2X-active and 1X-active nuclei in a population of fibroblasts from the same female.

An alternative interpretation is that control of X inactivation is exerted by down-regulation of transcription from the Xi in every cell, but this low level of transcription is not detected by RNA-FISH. However, we consider that this is unlikely because RNA-FISH detects transcription in nearly 100% of loci in male cells, and DNA-FISH detects two loci in nearly all female cells. Indeed, RNA-FISH is more sensitive than DNA-FISH, in which single molecules can be detected in interphase nuclei.

Moreover, we found that genes located close together on the Xi were usually expressed at different frequencies, and in the proportions expected of independent escape from inactivation. This implies that the probabilities of transcription of different loci on the inactive X are independently regulated.

We therefore propose that regulation of escape from XCI in marsupials amounts to the control of the probability of expression of a locus on Xi, rather than of the amount of expression from the locus. Thus, expression from genes on the inactive marsupial X is under a previously unsuspected type of epigenetic control, perhaps involving locus-specific regulatory factors causing local or regional changes in chromatin organization that determine the probability that a gene on the paternal X is transcribed.

This stochastic regulation of marsupial XCI seems to be quite different from the control of XCI in mouse and human. However, although the molecular aspects of XCI have been studied in detail for the past 50 years, no comparable RNA-FISH data have been published for XCI in eutherians, and it remains possible that escape of genes on the human inactive X is stochastic. It would be very instructive to study the cell distribution of 1X- and 2X-active nuclei for genes that partially escape inactivation on the human X.

### Inactivation of the marsupial X shows no polarity from an inactivation center

We constructed an activity map of the tammar wallaby inactive (presumably paternal) X in order to determine whether there was a polarity in frequency of expression. We observed no correlation between gene location and the frequency with which the allele on the Xi was expressed. Thus, there is no evidence of the polarity that was hypothesized [19] to reveal an inactivation center from which whole X chromosome control could emanate. Genes that are largely inactive were not clustered, nor were genes that largely escaped inactivation.

In addition, we found no correlation between Y expression and dosage compensation of the X paralogues. The highest frequency of escape was observed for *ATRX *(60%) and the lowest for *RBMX *(7%), both genes with Y paralogues that are not expressed in fibroblasts

RNA-FISH has the advantage that it provides information about individual cells; however, it is not quantitative, and intensity of signal does not correlate with expression level. Independent studies on marsupial Y-borne genes using qPCR show that Y paralogues either show testis-specific expression or are expressed much more weakly than their X partners [35,36] (Murtagh VJ, Sankovic N, Delbridge ML, Kuroki Y, Boore JL, Toyoda A, Jordan KS, Pask AJ, Renfree MB, Fujiyama A, Graves JAM & Waters PD, submitted).

These different expression profiles of X- and Y-borne paralogues, together with low X-Y sequence conservation (Murtagh VJ, Sankovic N, Delbridge ML, Kuroki Y, Boore JL, Toyoda A, Jordan KS, Pask AJ, Renfree MB, Fujiyama A, Graves JAM & Waters PD, submitted), suggests that Y genes have either a different or a diminished function compared with that of their X partners. Thus, the escape of these genes from XCI is unlikely to be the result of complementation by an active Y locus.

Indeed, the only feature that unites marsupial X genes with a high frequency of escape from X inactivation is that their human orthologues are located together on Xq22. Perhaps this reflects their original arrangement on an ancestral therian X 145 million years ago, at a position in which Y degradation occurred later and, therefore, XCI remains less complete.

Thus, marsupial XCI is controlled in a manner quite unlike that of the human and mouse X. In eutherians, XCI is a whole X phenomenon, in which activity domains are coordinately controlled by an inactivation center that contains the *XIST *gene. The independent control of the expression of loci on the inactive X is consistent with the absence of an *XIST *gene from the marsupial X [23,24,37].

### Tolerance to dosage differences

XCI is widely regarded as a vital mechanism that ensures proper dosage compensation between XY males and XX females, and the initial results from older studies of XCI in humans and mice indicated that, with rare exceptions, genes on the Xi were completely inactive. This strict adherence to dosage equivalence is consistent with observations of the disastrous effects of monosomies of an autosome or autosomal region in human patients. It may therefore seem surprising that dosage compensation for many X-borne loci is incomplete or absent in marsupial fibroblasts.

However, we now know that many genes on the human X chromosome escape from inactivation [38], particularly on the short arm, which was a relatively recent addition to the X and Y chromosomes [39-41]. Even on the mouse X, which seems to represent a state of near-complete inactivation, some genes are expressed from the Xi. The first genes on the human X that were shown to be 2X-active were those that retained partners on the Y chromosome [42], suggesting that their Y partners are (or were until recently) active and complement the function of the X genes, which therefore have no need of dosage compensation. Indeed, some of the genes we studied with paralogues on the Y chromosome do escape XCI on the marsupial X (*ATRX*, *UBA1*); however, at least some Y paralogues (for example, *ATRY*) are testis specific and do not complement. In addition, other marsupial X genes with a Y partner, such as *RBMX*, *PHF6X *and *HUWE1X*, do not escape inactivation.

Perhaps, then, dosage compensation is not as critical to development and function as we had supposed. This conclusion is supported by the recent evidence that the bird Z chromosome is compensated only partially, the 934 genes on the Z showing a range of male:female dosage relationships between 1.0 and 2.0 [4,43], and the demonstration that the five X chromosomes of the platypus (related to the bird Z and together representing more than 12% of the genome) seem to share this characteristic.

It may be that genes that require full compensation are especially sensitive to dosage effects because changes in their dose propagate through numerous downstream gene networks. Dosage differences in some genes may be critical for development of sexual differences, as is the case for the *DMRT1 *gene in birds [44]. In contrast, non-compensated genes may participate in intracellular housekeeping and catalytic activities that are regulated at many other levels, so their function is less sensitive to gene dosage. Such ubiquitously expressed genes are over-represented in the list of marsupial genes that largely escape inactivation.

We propose here that, during sex chromosome differentiation, the gradual loss of genes from the proto-Y chromosome selected for inactivation of the paternal allele of the homologous X-borne genes that were particularly sensitive to dosage differences in one tissue or another. This resulted in piecemeal inactivation that was tissue specific, as is observed for marsupial XCI. We suggest that the cooperative nature of the chromatin changes recruited to silence this locus in eutherians involved non-critical loci nearby. This spreading of inactivation from dosage-sensitive loci is almost complete in mouse, but has left many escaping gaps in the human X, especially on the recently recruited short arm.

### Evolution of X chromosome inactivation

The fundamental difference between marsupial and eutherian XCI led us to look for similarities with dosage compensation in more distantly related mammals and non-mammal vertebrates. Indeed, the stochastic inactivation we observed in marsupials is similar to that we described recently for genes on the five X chromosomes of the platypus. X-specific genes are expressed from one or both alleles in different fibroblasts from the same female, and the frequency of 1X-active and 2X-active nuclei is a consistent feature of each gene, ranging between 20% and 53% of 2X-active nuclei [7]. However, it is hard to impute an evolutionary link between monotreme and marsupial dosage compensation since platypus X chromosomes have no homology with those of marsupials and eutherians; rather, they share considerable homology with the Z chromosome of birds [10]. Dosage compensation in the chicken is known to be incomplete, ranging from a ZZ male:ZW female ratio of 1.0 to 2.0 for different genes [4]. Limited RNA-FISH was reported for five genes [5], but the low efficiency of detection makes it difficult to assess whether differences in expression represent a down-regulation in each cell, or a stochastic control of expression.

Perhaps, then, marsupial XCI retains features of an ancient silencing mechanism common to all chromosomes. The stochastic nature of marsupial and monotreme X chromosome expression is reminiscent of monoallelic expression from many autosomal loci, including olfactory receptors and immune genes such as immunoglobulins, T-cell receptors and natural-killer-cell receptors [45]. It is tempting to speculate that this reveals an ancient mechanism to control gene expression, which was exapted to evolve into an X chromosome compensation system independently in monotremes and therians [46].

A stochastic basis for transcriptional activation can be seen as a sequence of events that combines a random element, such as transcription factor binding, with a selective step, such as cell commitment. For example, a 'probability-promoting factor' identified in mouse tetraploid cells allows each X chromosome to independently determine the probability of initiating XCI [47]. The probability of inactivation of one or other X chromosome in mouse can be altered by mutations in a locus near *XIST *[48]. The inactivation of a single X is locked in by a feedback mechanism, controlled by the XCI center, which suppresses the inactivation of the active X [49]. Stochastic allelic expression of genes gives rise to a diverse repertoire of cells and creates diversity, so although individual cell expression profiles vary, even within a clone, the net result for a cell population will be a stable outcome.

Did an ancestral paternal, stochastic, and incomplete inactivation system, still represented by marsupials, evolve into the hyperstable chromosome-wide inactivation of eutherian mammals? The similarities of marsupial XCI with the first wave of XCI in the extraembryonic tissue of rodents and bovine (which is also paternal, incomplete and methylation independent) suggests that this represents the inactivation system in an ancient therian mammal, and it underwent changes to render it more complete and stable in eutherians. It will be very interesting to discover whether XCI in mouse embryonic membranes is, like marsupial XCI, locus specific and stochastic.

How did XCI evolve into a whole-chromosome system? The evolution of the *XIST *gene early in the eutherian lineage, perhaps by insertion of repetitive sequence [24] and pseudogenization of an ancient tetrapod gene[37], brought neighboring inactivation domains under chromosome-wide control. Binding with *XIST *RNA permitted the binding of modified histones and made DNA methylation more probable, resulting in stabilization of inactivation. Perhaps, then, stochastic expression is also the basis of random inactivation in the embryo of eutherian mammals.

## Conclusions

We found that genes on the tammar wallaby X chromosomes are dosage compensated to different extents. In marsupials XCI is incomplete and locus specific, and escape from inactivation occurs independently on a gene-by-gene basis. The frequency of escape is not related to the presence or absence of a Y-borne paralogue, and does not depend on gene location. This is unlike the clustering of genes that escape inactivation on the region of the short arm of the human X that was added to the ancient X, and became subject to inactivation only recently. Marsupial XCI is best explained by control of the probability of expression of a paternal allele in different nuclei, rather than of the amount of expression. This suggests a stochastic basis for XCI in marsupials, similar to that observed for platypus (and perhaps bird) dosage compensation, and raises the possibility that dosage compensation of sex chromosomes evolved from an ancient system of stochastic monoallelic expression observed for many autosomal genes.

## Materials and methods

### qRT-PCR

RNA was extracted from five male and six female tammar wallaby fibroblast cell lines with a GenElute™ Mammalian Total RNA Miniprep Kit (Sigma, Castle Hill, NSW Australia) according to the manufacturer's instructions. Reverse transcriptions were conducted with SuperScript™ III First-Strand Synthesis System for RT-PCR (Invitrogen, Carlsbad, CA, USA) according the manufacturer's instructions.

Primers (Additional file 3) for X/Y shared genes, X-borne genes, and the control gene were designed following the QuantiTect^® ^SYBR^® ^Green PCR Handbook (QIAGEN, Doncaster, VIC, Australia)). All primer pairs were tested on male and female genomic DNA and they all generated the single PCR products of the expected size for each template. The identity of the PCR products was confirmed by direct sequencing. All qPCR reactions were set up in triplicate with the QuantiTect^® ^SYBR^® ^Green PCR system, and amplifications were performed and detected in a Rotorgene 3000 cycler (Corbett Research, Doncaster, VIC, Australia). Cycling conditions were as follows: 15 minutes at 95°C; followed by 45 cycles of 94°C, 15 minutes at 58°C, 20 minutes at 72°C; followed by a 55°C to 99°C melt analysis to check product specificity. Expression levels of test genes relative to *GAPDH *in each tissue were calculated using the comparative quantification software supplied by Rotorgene.

### Cell culture and RNA-FISH

Male and female fibroblast cell lines were cultured on 0.1% gelatin-coated coverslips in AmnioMax C100 medium (Invitrogen) at 35°C in 5% CO_2 _to a density of 60 to 80%. The cells were rinsed in RNase-free 1× phosphate-buffered saline, and then permeabilized in fresh CSK buffer (100 mM NaCl, 300 mM sucrose, 10 mM PIPES pH 6.8)/0.5% Triton X 100/2 mM Vanadyl Ribonucleoside Complex (Sigma, Castle Hill, NSW Australia) for 8 to 10 minutes on ice. Cells were then fixed in fresh 3% paraformaldehyde/1× phosphate-buffered saline for 10 minutes at room temperature. Coverslips were then washed twice for 5 minutes in 70% ethanol, and stored for up to 2 months in 70% ethanol at -20°C. Just prior to RNA-FISH experiments, the coverslips were dehydrated in 80% ethanol, 95% ethanol and 100% ethanol for 3 minutes each and air-dried.

BACs or fosmids containing the genes of interest are from three different genomic libraries: Me_KBa, Arizona Genomics Institute, Tucson, AZ, USA; Me_VIA, Victorian Institute of Animal Science, Attwood, VIC, Australia tammar BAC libraries; and MEFX, Tammar wallaby X chromosome specific fosmid library. Probes [20] were labeled in a nick translation reaction with either biotin-16-dUTP or digoxygenin-11-dUTP (Roche Diagnostics, Indianapolis, IN, USA), Spectrum-Orange or Spectrum-Green (Abbott Australasia Pty Ltd., Botany, NSW, Australia). Unincorporated nucleotides were removed from labeled probes using Probe-Quant G50 micro Columns (GE Healthcare, Chalfont, Buckinghamshire, UK). Probes of a test gene and control gene were co-precipitated with 20 μg of glycogen and 1 μg tammar wallaby C_0_t1 DNA. The air-dried pellet was resuspended in 5 μl of formamide and then denatured at 75°C for 7 minutes. Following transfer to ice, 5 μl of 2× hybridization buffer (4× SSC, 40% dextran sulfate, 2 mg/ml bovine serum albumin, 10 mM vanadyl ribonucleoside complex) was added to each probe, which were then pre-annealed at 37°C for 20 minutes. Ten microliters of probe was added immediately to the coverslip for overnight hybridization at 37°C.

After hybridization, coverslips were washed three times for 5 minutes each in 50% formamide/2× SSC at 42°C, and three times more for 5 minutes each in 2× SSC at 42°C. Coverslips were incubated in blocking buffer (4× SSC/0.1% Triton/5% bovine serum albumin) for 15 minutes at room temperature. Biotin-labeled probes were detected with avidin-FITC (Vector Laboratories, Inc., Burlingame, CA, U.S), with FITC signals amplified by additional layers of biotinylated anti-avidin (Vector Laboratories, Inc., Burlingame, CA, USA) and avidin-FITC. Coverslips were incubated with the primary antibody in blocking buffer for 40 minutes. Coverslips were washed three times in 2× SSC for 5 minutes each, followed by incubation and washing of the secondary antibody under the same conditions as the primary antibody. Coverslips were mounted in Vectashield^® ^with DAPI (Vector Laboratories, Inc., Burlingame, CA, USA).

Nuclei were viewed and RNA signal was detected using a Zeiss Axioplan2 epifluorescene microscope. Images were collected and merged using a SPOT RT Monochrome CCD (charge-coupled device) camera (Diagnostic Instruments Inc., Sterling Heights, MI, USA) and IP Lab imaging software (Scanalytics, Inc., Fairfax, VA, USA).

RNA-DNA FISH was performed with modification of a published technique [50]. For overlaying DNA-FISH, coverslips were fixed, dehydrated, denatured, dehydrated again and hybridized at 37°C overnight to DNA probes labeled opposite (for example, spectrum green versus spectrum orange) of the RNA label. Coverslips were washed stringently and probe was detected as above.

Efficiency of RNA-FISH hybridization was determined from the results obtained in male fibroblasts and extrapolated to determine the expected frequency of nuclei with two signals, one signal and no signal per cell using the formula p^2 ^+ 2pq + q^2 ^= 1, where p^2 ^is the number of nuclei with two signals, 2pq (q = 1 - p) represents nuclei with one signal and q^2 ^is the number with no signal. *P*-values were determined by a χ^2 ^test with two degrees of freedom.

## Abbreviations

BAC: bacterial artificial chromosome; FISH: fluorescence *in situ *hybridization; qPCR: quantitative PCR; Xa: active X chromosome; XCI: X chromosome inactivation; Xi: inactive X chromosome; XIST: X inactive specific transcript.

## Authors' contributions

SAN, EK and PDW performed the RNA-FISH experiments. SAN, KSJ and PDW performed the expression analysis. SAN drafted the manuscript. JAMG and EK conceived the study. JAMG, JED and PDW contributed to the design and coordination of the study and were involved in the preparation and revision of the manuscript. All authors read and approved the final manuscript.

## Supplementary Material

Additional file 1 **Male and female gene expression for 13 ubiquitously expressed genes on the tammar wallaby X chromosome**. Genes are presented in the order in which they are located on the X, from the centromere down. No expression was detected for *PLP1 *in male or female fibroblasts, so this gene was eliminated from the analysis. Expression of these genes in fibroblast cell lines (five males and six females) was normalized to the expression levels of the autosomal housekeeping gene *GAPDH*. For all but two genes (*G6PD *and *TBC1D25*), a higher level of expression was consistently observed in females over that in males. A high variability between individuals was observed that could not be attributed to particular cell lines consistently showing higher or lower expression for all the genes tested. This variability between individuals is thought to reflect differences in the rate of transcription, but could equally well reflect differences in the probability that a locus is transcribed. Click here for file

Additional file 2 **RNA-FISH results for two additional females and two males cell lines**. Click here for file

Additional file 3 **List of primer pairs used for qRT-PCR**. Click here for file
