# Supplementary material for: Activity map of the tammar X chromosome shows that marsupial X inactivation is incomplete and escape is stochastic
Source: Genome Biol. 2010 Dec 23;11(12):R122. doi: 10.1186/gb-2010-11-12-r122 (PMC3046482; doi:10.1186/gb-2010-11-12-r122)
Supplement: Additional file 1 — Male and female gene expression for 13 ubiquitously expressed genes on the tammar wallaby X chromosome. Genes are presented in the order in which they are located on the X, from the centromere down. No expression was detected for PLP1 in male or female fibroblasts, so this gene was eliminated from the analysis. Expression of these genes in fibroblast cell lines (five males and six females) was normalized to the expression levels of the autosomal housekeeping gene GAPDH. For all but two genes (G6PD and TBC1D25), a higher level of expression was consistently observed in females over that in males. A high variability between individuals was observed that could not be attributed to particular cell lines consistently showing higher or lower expression for all the genes tested. This variability between individuals is thought to reflect differences in the rate of transcription, but could equally well reflect differences in the probability that a locus is transcribed. [file gb-2010-11-12-r122-S1.docx]

**DC status Complete Partial Absent**

**F:M ratio 0 1 2 3**

*
