# Supplementary material for: Activity map of the tammar X chromosome shows that marsupial X inactivation is incomplete and escape is stochastic
Source: Genome Biol. 2010 Dec 23;11(12):R122. doi: 10.1186/gb-2010-11-12-r122 (PMC3046482; doi:10.1186/gb-2010-11-12-r122)
Supplement: Additional file 2 — RNA-FISH results for two additional females and two males cell lines. [file gb-2010-11-12-r122-S2.doc]

## Additional file 2 – RNA-FISH results for two additional females and two males cell lines.

| **Genes on BACs or fosmids** | **Percent cells with** | | | | | | | | |
| --- | --- | --- | --- | --- | --- | --- | --- | --- | --- |
|  | **1 signal** | | **2 signals** | **1 signal** | **0 signal** | **2 signals** | | **1 signal** | **0 signal** |
| *MECP2X, IRAK1, TMEM187* | 96 | 99 | 42 | 49 | 9 | 57 | | 42 | 1 |
| *ATRX* | 97 | 99 | 57 | 39 | 4 | 48 | | 44 | 8 |
| *UBA1, RBM10* | 97 | 98 | 66 | 31 | 3 | 59 | | 40 | 1 |
| *TBCD125, GATA1* | 96 | 95 | 18 | 80 | 2 | 11 | | 84 | 5 |
| **Cell line** | **Male** | | **Female** | | | | | | |
|  | **A** | **B** | **C** | | | | **D** | | |
