# Supplementary material for: Activity map of the tammar X chromosome shows that marsupial X inactivation is incomplete and escape is stochastic
Source: Genome Biol. 2010 Dec 23;11(12):R122. doi: 10.1186/gb-2010-11-12-r122 (PMC3046482; doi:10.1186/gb-2010-11-12-r122)
Supplement: Additional file 3 — List of primer pairs used for qRT-PCR. [file gb-2010-11-12-r122-S3.doc]

**Additional file 3 – List of primer pairs used for qRT-PCR.**

| **Primer Set** | **Sense primer 5' - 3'** | **Antisense primer 5' - 3'** |
| --- | --- | --- |
| *RBMX* | TGGGGGTCCTCCACCTAAAAG | TGGCATTGGGTCTCTGCGTG |
| *ATRX* | AAATCCAATCCAGAATGGTCAGTG | TGGTGGCAGGAACTTTGTTAGG |
| *PHF6X* | GAACTGGAGCCTTCATCACC | TGGGACTGCTTCTGTGTGG |
| *HUWE1X* | GACGCAAGCACACCGAGAAG | GCTAACTGGTTGAGGGTATCCAAG |
| *UBA1* | CGGAAAAGTCCATCCCCATCTG | TCTGCTGGCTGCTTGAAGAGACC |
| *MECP2X* | AAACTCAAGCAGAGGAAATCAG | CGAAGTATGCAATCAACTCTACC |
| *PLP1* | ACATCAAGCTCACTCTTTGGAC | TATGTAGACAGGCACAGCAGAG |
| *G6PD* | TCAAACCTCACTGTGGATGAC | CACGTAGGAATTTCGAGAGAAG |
| *GLA* | ACGAGAATGGTAGGCTCAAG | GTACCCAGCACAGGTGAGAG |
| *AR* | TGCCCATTGACTATTACTTTCC | TACTTCTGTTTCCCTTCTGCTG |
| *PLP1* | ACATCAAGCTCACTCTTTGGAC | TATGTAGACAGGCACAGCAGAG |
| *UPF3B* | AACCAGGAGGACATTGTTTTG | AAAGGAGCGAATTCCACTATG |
| *GAPDH* | AAGTTCAAGGGCACTGTCAAGG | GACTCTACAACATACTCGGCTCCA |
